# Society Reports—Medico-Chirurgical Association

**Published:** 1879-04-20

**Authors:** 


### SOCIETY REPORTS.

### *MEDICO-CHIRURGICAL ASSOCIATION.*

*Atlanta, Ga., March 29th*, 1879.

President POWELL in the Chair.

Dr. R. C. WORD reported the following case, asking the views of members as to the diagnosis.

I submit the following notes of a case which occurred in my practice, the diagnosis of which I confess myself at some loss to determine.

Mr. H., after severe exposure to rain and cold was seized, on Friday, December 19th, with a chill which lasted two or three hours, followed by high febrile reaction, severe pain in the head and back, aching in the limbs, etc., with intense thirst. The friends gave him Tutt's pills and bone-set tea, but the fever continued with increasing violence until the time of our visit, which was on the morning of the 20th, at four o'clock. We found him with the above symptoms; the pulse at 105, full and tolerably firm. Supposing it to be the commencement of a febrile attack we gave a dovers powder, applied cold applications to head, and ordered five drops tincture veratrum every two hours until pulse falls to 75, then to be continued in smaller doses, to keep up the impression, until next visit.

December 21st, 10 o'clock a.m. Patient had rested some better during the night; pulse had once fallen to 70, but had risen since daylight, and is now at 100, full and occasionally intermittent, but softer than at first visit. There are symptoms of cerebral congestion; intense headache; eyes red; considerable nervous prostration; is delirious, except when fully aroused. Complains of his throat, which upon examination is found red and swollen, and the palatine arches besprinkled with an eruption very like that of measles. During the night there had been several copious discharges from the bowels, of a thin, light brown appearance. Prescribed a dovers powder to restrain the bowels, and ordered veratrum 5 drops; bromide potassium 10 grs. every two hours. Cold applications to the head, blisters to temples and back of ears, warm pediluvia. After about two hours a rash resembling measles appeared upon the neck, chest and forehead and soon spread over the entire body. On the sides and back, the eruption was blended with urticaria and a few minute petecchial spots, and soon the rash extended over the entire surface, very much like measles at its full hight. The eyes were red, yet there was wanting the cough and the characteristic semi-lunar interspaces of the measle eruption, nor did it answer fully to the eruption of scarlatina. It more closely resembled measles than anything else, and as the patient had never had that disease and had visited a village where it was prevailing, I decided that it was measles, aggravated by exposure to cold, etc.

At this stage of the case a medical friend in the vicinity, Dr. Fowler, saw the patient with me. It was decided to use remedies calculated to encourage the eruption. Warmth to the lower extremities with frictions to the body, and small doses of pulvis doveri occasionally, and when the eruption was fully out, small doses of aconite often repeated. Head to be kept cool.

Dec. 22d, 9 a. m. Found patient in a comatose state; pulse weak and rapid; rash somewhat suppressed; the skin not so red. Ordered cold water freely to scalp; stimulating frictions to the entire body, and heat to the extremities, but with no impression. The patient rapidly failed, and died about six o'clock the same evening.

While this case was regarded as one of malignant measles, yet there were children in the family who had not had measles and who *did not contract* the disease from this patient.

A brother practitioner had a similar case in the same neighborhood, having the same sort of eruption, and which likewise proved rapidly fatal. I also treated another case in the vicinity, near the same time, which was not unlike the above, the eruption, however, being absent. There being some retraction of the head and stiffness of the muscles of the neck in this latter case, we pronounced it cerebro-spinal meningitis; it recovered under revulsives, bromide of potass. and opiates. Is it not probable that the other two cases were cases of cerebro-spinal meningitis, of the spotted fever type?

Dr. W. T. Goldsmith remarked that he had observed no petecchial spots or eruption in any case he had seen in this country. He considered Dr. Word's case an eruptive fever, most probably malignant measles, as at first diagnosed by him. There was wanting the retraction and rigidity of the head and neck, which is pathognomonic of cerebro-spinal meningitis.

Dr. A. J. Pinson said he did not regard the case reported as cerebro-spinal meningitis. He had seen many cases of the disease, and the description does not apply to the affection as he had observed it.

Dr. A. R. Alley remarked that he had seen many cases of meningitis, in two of which he had noticed an eruption on the skin. In all the cases, however, there was a white fur on the tongue, and rigidity of the muscles of the neck. In one case, in a child, there was an eruption, opisthotonos and speedy death. He thought such cases allied to typhus. Some regarded the disease as rheumatic in its origin. He had witnessed a number of post-mortem examinations and had observed that the gray matter of the brain, the seat of reflex action, was involved, with softening and a striated appearance of the meninges, and frequently a pultaceous broken down pus in the convolutions and ventricles. The same conditions sometimes extended to the spinal cord. He thinks there was evidence also of an increase of fibrin in the blood. In some cases there was effusion and rheumatic irritation about the joints. He thinks Dr. Word's case was one of malignant measles.

Dr. Thomas S. Powell said he did not think the case reported was measles, nor could he say that it was cerebro-spinal meningitis. The eruption did not answer fully to that of measles; it was mixed and wanting in some of the more striking characteristics of that disease. Eruptions of the skin are not an unfrequent accompaniment of different diseases, and may depend upon a variety of causes. The fatal result in the case was doubtless due to congestion or inflammation of the brain.

In many instances cerebro-spinal meningitis, according to his experience, is caused by blood poison produced from the use of stale, tainted or corrupt food. Exposure to miasma and sudden atmospheric vicissitudes may be an exciting cause, but the poison of bad food is well calculated, and he was certain predisposed the system to the malady. He mentioned three cases in his practice which confirmed this view. One, a boy 13 years old, whose father kept a provision store wherein tainted vegetables and other stale supplies were kept and consumed by the family. The case was well marked and attended with retraction of neck, opisthotonos, etc., It recovered, though not fully, the mind being impaired, and locomotion not perfect after a period of nearly two years.

Another case was that of a negro boy at a saloon, who indulged freely in bad articles of diet, and had an attack very like the above.

A third case was a white boy, at a country store, where the tainted food and the same attendant circumstances were present as in the other two cases. He treated all the cases with large doses of quinine and counter irritants. It is now conceded that the blind staggers in the horse is caused by feeding on rotten and smut corn. The brain may be affected by these substances. As to eruptions, they are known frequently to be sympathetic from the stomach, as urticaria from eating pickles, fish, etc. Many cases of so-called roseola and scarlatina of our spring seasons are but sympathetic eruptions from the use of unripe or crude fruits of the season. Such cases he treats with chlorate of potash and alkalies, and they recover.

Dr. Pinson stated that he could not accept the theory of Dr. Powell, as to bad food being the cause of cerebro-spinal meningitis. During the war he had seen the disease in a fatal form attack the troops at Charleston, at a time when they were well supplied with wholesome food, while in Virginia it did not prevail; had also seen cases of the disease in vicinity of Cave Spring, Ga., about six years ago. There were 10 deaths from it. These cases were near a mill pond, and may have resulted from malaria.

Dr. Powell replied that the statements of Dr. Pinson tended to confirm rather than to refute his position. In Charleston, the climate is hot, and the atmospheric conditions such as are likely to damage deposits of food as we find them in commissary quarters and stores in large cities. In our cities generally, and sometimes in villages, there is bad food and bad cooking found. Dr. Pinson's mill cases may have resulted from the meal of rotten corn obtained at the mill. Now-a-days the shelling of corn is mostly done by machinery, and it is estimated that about ⅛ of it thus shelled is rotten or unsound. Some years ago a large lot of damaged corn was bought and the meal from it sold by one of our merchants at reduced rates, and the result was a good deal of sickness among the people. In one instance, a mule fed on it died with the blind staggers in a short while after eating it. Dr. Pinson also said the disease did not at that time prevail among the Virginia troops. Of course the soldiers did not have meningitis in Virginia; in that country they have good food and the finest cooks in the world. "Nubbing" the corn before shelling is a word very common, and well understood by every household in the "old dominion." It consists in first shelling off and throwing aside the defective grains on the small end of the cob, and grinding only the sound corn.

In former times, when the people of every section raised their own supplies, this disease, as well as diphtheria and like affections were scarcely known; but in these days of railroads and steamboats, when food is transported over long lines from one section to another, it often undergoes changes which, though not always perceptible, affect its purity or wholesomeness, and lead to various diseases among which, in my judgment, is cerebro-spinal meningitis.

The importance of sound food is no new question. Most of our larger cities are provided with food inspectors, and strict market regulations are required; but in other places, especially in the South, the indifference and carelessness upon this point is almost criminal. Good food, pure food, and a plenty of it, is essential to health, mental and physical. Food makes both muscle and brain. If you want great men, men with both physical and mental endowments, capable of great achievements, and exempt from meningitis and its kindred diseases, feed them well from their birth up. Close observation will show that the great men of every country, as a rule, are raised in certain cities or localities where food, climate and surroundings are favorable to the largest physical and mental development.

The laws of nutrition and the physical economy provide for the fœtus in utero and at the breast. As it develops to youth and becomes an independent being, the law, or principle, which at first supplied the needs of the system should be still observed and continued, and such food provided as will best develop the mental and physical man; hence the importance of the food question, and its bearing upon health and disease, especially in the class of affections we are discussing.

Dr. A. R. Alley said he could not agree with Dr. Powell. He had resided in Charleston and could say that her people live well and eat very little corn. Pathology shows that bad food is not the cause of cerebro-spinal meningitis. Bad food may cause nausea, vomiting and various affections, but not meningitis. Nor is the blind staggers in the horse and cerebro-spinal meningitis the same affection.

Dr. E. J. Roach said he did not know what Dr. Word's case was, but did not think it was cerebro-spinal meningitis. There was no opisthotonos, which is characteristie of this disease. He thought that bad food, as suggested by Dr. Powell, was often a cause of the disease, though perhaps only an exciting cause. He thought that atmospheric changes and malaria had much to do in developing the affection.

Dr. Goldsmith reaffirmed his position that the case was measles. The eruption in the throat was indicative of measles. It is known that the eruption is often visible in the throat before it appears on the external skin, and in exceptional cases, both in measles and scarlet fever, the eruption is entirely wanting. Such cases are apt to be fatal. He thought that cerebro-spinal meningitis was due to blood poison and should be classed with the zymotic affections. Bad food may prepare the way, perhaps, for this, as with many other diseases, but is certainly not alone the cause of the affection.

Dr. J. J. Knott remarked that it was well known that his views of cerebro-spinal meningitis were different from those of the profession generally. He thought bad food had nothing whatever to do with it; on the contrary, those best nourished were more liable to it. Healthy, vigorous children, and youth from 7 to 15 years were most susceptible. Females rarely have it; probably because less exposed. The seasons have much to do in causing the disease, particularly the prevalence of east winds. The symptoms are more varied than is usually represented; opisthotonos is frequent but not present in every case; many of the worst cases do not have this symptom. The atmospheric impression is made upon the ganglionic and vaso-motor systems. There is often hyperæsthesia, showing a change in the afferent and efferent nerves. If you impress or partially paralyze a nerve you change the electro-tonic condition. There is first irritation, then congestion, then inflammation. If we wait until the inflammatory stage appears no remedy can avail. The best treatment, and one which had proven most successful, was the early administration of large and repeated doses of quinine, with turpentine applied to the spine by means of a hot iron upon flannel. Cases could be relieved by this method in a few hours. The quinine should be continued for three or four days to guard against relapse.

Dr. Goldsmith remarked that if the state of the atmosphere or East winds bring about the disease it should always follow when the same atmospheric conditions are present, which is not the case, there being often long intervals of time when the disease is absent; yet the atmospheric conditions are present every year.

Dr. Knott stated that the disease is present in greater or less degree every year.

Dr. Goldsmith remarked that the disease is generally regarded as of recent origin; yet the East winds had always prevailed, and the same fact may be urged against the bad corn or bad food theory of Dr. Powell.

The same causes should always produce the same results, so that when the effect is experienced we could find the cause. When we see a sudden rise in the river or streams about us, and no rain, we know that there has been a flood of rain above us. So should it be in regard to the cause of disease. The truth is that in this disease, as with influenza and other epidemics, the cause is not yet known, for we have scarcely yet entered upon the threshold of discovery.

Dr. Knott replied that whether his theory was correct or not, the treatment based upon the theory had been successful, not only in his own hands, but with many others. As to the remote cause, it was probably the absence of ozone in the atmosphere.

Dr. Alley said that in Augusta, in the year 1872, cerebro-spinal meningitis prevailed both among the negroes and among the well-to-do and refined families in the city.

Dr. Powell said that he was misunderstood; he did not contend that bad food alone caused the disease. It may be true in the abstract that many diseases have a remote, unknown cause, yet the system may not be susceptible, or may resist the cause. The predisposition must first obtain, and whatever predisposes or renders the system liable may be regarded as a cause.

---

**Hypodermic Injections of Tincture of Ergot for Retention of Urine.**—Mr. Luton, of Rheims, employs a mixture of one part of tincture of ergot in five parts of alcohol at 90°, by hypodermic injection, in the treatment of inorganic retention of urine, The dose he employs is from seven and a half to thirty drops, fifteen drops of the solution being equal to three grains of powdered ergot. He has used it in the paralysis of the bladder accompanying typhus, confluent variola, and acute hydrocephalus. He makes the injection in the fossa behind the great trochanter. Within half an hour, and sometimes within a few minutes, a complete and forcible evacuation of the bladder takes place. He has never observed an eschar of the skin or a gangrenous abscess after the injection.—*Le Lyon Medical.*

